# Teacher Support and Pre-Service Preschool Teachers’ Piano Skill: The Chain Mediation Effects of Music Self-Efficacy and Learning Engagement

**DOI:** 10.3390/bs15040484

**Published:** 2025-04-07

**Authors:** Tingjun You, Huihua He, Yuanyang Yue

**Affiliations:** 1 Shanghai Institute of Early Childhood Education, Shanghai Normal University, Shanghai 200234, China; youtingjun@shnu.edu.cn; 2 Research Center of Lifelong Education Policy, Shanghai Normal University, Shanghai 200234, China; 3 School of Education, Shanghai Normal University Tianhua College, Shanghai 201815, China; yyy2138@sthu.edu.cn

**Keywords:** pre-service preschool teachers, teacher support, music self-efficacy, learning engagement, level of piano skill

## Abstract

Piano proficiency is essential for pre-service preschool educators; nevertheless, numerous candidates begin teacher education programs with little prior experience, exhibiting poor self-efficacy and limited musical competencies required for effective teaching. This study seeks to examine the mechanism of how teacher support affects proficiency in piano skills among pre-service preschool educators. Four hundred thirty pre-service preschool teachers from a preschool teacher education program at a public normal university in Shanghai, China, completed the Teacher Support Questionnaire (MOCSE-TSQ), Attitudes Toward Musical Activities and Performance, and the Classroom Engagement Inventory in Music (CEI-M). The findings revealed a substantial positive link between teacher support and the proficiency in piano skills among pre-service preschool educators. Teacher support positively predicted piano ability levels via the chain-mediated effects of musical self-efficacy and learning engagement among pre-service preschool educators. The “Support–Feedback–Reflection” (SFR) cyclical development model was employed as an approach for improving the musical abilities of pre-service preschool teachers. These findings provide empirical information to guide curriculum development and pedagogical enhancements for piano courses targeting pre-service preschool educators.

## 1. Introduction

Piano playing could be considered not only as a teaching method in early music curriculum, but also a pedagogical strategy to attract young children’s attention, provide opportunities for teachers to interact with children more effectively and relieve anxiety level of children with special needs. Piano performance is not only a form of instrumental performance, but also, with its unique melody and dynamic expressiveness, can be applied to other musical activities (Han & Chen, 2017; Rakhimova, 2024). In an early childhood education setting, research on kindergartners showed that piano accompaniment positively influenced kindergartners’ singing abilities over a year of music instruction, highlighting its role in early music education (Atterbury & Silcox, 1993). Evidence shows that children exposed to piano-accompanied music-movement activities showed marked improvement in rhythmic synchronization and auditory discrimination (Laure & Habe, 2024).

The use of piano-assisted music activities not only helps to enhance musical abilities, but also has a positive effect on the development of other aspects of young children. Previous study shows that piano instruction improves children’s working memory (Price-Mohr & Price, 2021) and speech perception (Nan et al., 2018). Piano-accompanied musical activities promoted children’s learning engagement and social interaction (Zhang, 2024). A study found that after eight weeks of piano-assisted music activity intervention, the experimental group of preschool children showed significant improvement in early mathematical reasoning ability, confirming the effectiveness of such music activities in promoting preschool children’s math ability (Doğan & Tecimer, 2018). Focusing on piano instruction, it has important positive effects on the development for young children. Evidence shows that short-term piano instruction has been shown to improve working memory and word recognition in children. Piano training also enhances the neural processing of pitch, which is closely linked to improved speech perception (Nan et al., 2018) and executive functions in preschool children, such as emotional control, planning, and organization (Qui et al., 2013). Therefore, piano proficiency significantly benefits preschool teachers by improving their ability to teach music, engage children, and create a stimulating learning environment (Wan et al., 2025). This underscores the importance of incorporating piano training in early childhood education programs (Akgul, 2022; Li, 2012; Nakahira et al., 2010; Wan et al., 2025).

Many studies have focused on the music capabilities of preschool teachers (Ho Weatherly & Weatherly, 2024; Koutsoupidou, 2010; Rajan, 2017; Burak, 2019; Lee, 2009; Iwaguchi, 2012). For instance, the Ministry of Education of China issued the “Guidelines for Preschool Education (Trial)” (MOE, 2001) This guideline proposes artistic domains as an integral component of early childhood education, recognizing the role of arts in children’s holistic development and creative expression (MOE, 2001). The Chinese Ministry of Education also issued the “National Professional Standards for Kindergarten Teachers” (MOE, 2012b) and the “Guidelines for Learning and Development of Children Aged 3–6” (MOE, 2012a). These documents provide professional standards for preschool teachers and guidance on artistic activity goals. The “National Professional Standards for Kindergarten Teachers” proposes that preschool teachers should possess professional knowledge and skills in the field of art. Moreover, “the Guidelines for Learning and Development of Children Aged 3–6” suggested that classroom teachers have the responsibilities to support young children experience, appreciate, express, and be creative in various artistic activities implemented in their preschool and kindergarten.

In addition, to promote the high-quality development of preschool education, the Ministry of Education has launched the “Excellent Teacher Training Program” in 2014 (MOE, 2014), focusing on cultivating an excellent team of preschool teachers. In this context, music skills and music teaching methods courses have emerged as essential components of the curriculum systems in Chinese higher education and pre-service preschool teacher programs (Jing & Danprdit, 2022; Xin & Pattananon, 2022; Xue et al., 2024). Early childhood art education is one of the 11 specialized courses in all preschool teacher education programs (MOE, 2018). In Shanghai, education schools in universities have established full-time preschool education programs, which include piano lessons in their curriculum (East China Normal University, 2024; Shanghai Normal University, 2024). Generally, these piano courses use performance assessment as a primary evaluation method and are offered from the first year through the third year of undergraduate studies. The fourth-year curriculum primarily focuses on graduation internships (East China Normal University, 2024; Shanghai Normal University, 2024).

Preschool teachers’ music ability has garnered attention from scholars worldwide, with researchers from Australia, Croatia, America, and other countries. However, their researchers find that many preschool teachers face challenges when facilitating music activities due to their limited background in music experience and music skill learning (Bainger, 2010; Cvrtila, 2024; Kondracka-Szala & Michalak, 2019; Rajan, 2017). Furthermore, many pre-service preschool teachers enter teacher education programs with little to no prior experience in piano playing in Japan (Iwaguchi, 2012). Due to this limited background, many exhibit low levels of learning initiative, self-efficacy, and confidence in developing musical skills and organizing music activities for young children (Cvrtila, 2024; Hae & Kemple, 2011; Wei et al., 2024). Furthermore, a significant number of pre-service teachers acknowledge that their current musical abilities are insufficient for effective preschool music education (Ehrlin & Wallerstedt, 2014). While existing research highlights the role of external guidance in piano learning for pre-service preschool teachers (Adamyan, 2018; Zhengshan & Sondhiratna, 2024), less attention has been given to internal motivation and support mechanisms.

According to social support theory, both psychological and practical support enhance an individual’s capacity and motivation to engage in specific behaviors (House, 1971). When students feel respected, understood, and encouraged by their teachers, they are better equipped to overcome challenges and build confidence (Wentzel et al., 2010). Moreover, recognition and support from others contribute to higher self-efficacy, whereas a lack of such support can lead to psychological and behavioral difficulties (Van Rooij et al., 2017). Notably, research highlights that educators serve not only as organizers and facilitators of teaching activities (Orejudo et al., 2021) but also as key sources of internal support. Through their guidance, teachers can alleviate anxiety, enhance learning confidence, foster engagement, and improve musical achievement (McPherson & McCormick, 2000).

Therefore, in piano instruction, teachers should provide not only technical guidance but also emotional encouragement while allowing pre-service preschool teachers sufficient autonomy (Iwaguchi, 2012). Comprehensive teacher support can reduce psychological anxiety, enhance confidence and self-efficacy, foster a passion for learning, increase engagement, and ultimately improve piano skills. This study aims to examine the relationship between teacher support and piano skill level among pre-service preschool teachers, as well as the underlying mechanisms that mediate this relationship.

### 1.1. Teacher Support and Level of Piano Skill

The concept of teacher support (TS) originates from social support theory and educational psychology (Trickett & Moos, 1973). Tardy (1985) initially defined social support in educational contexts through four dimensions: emotional, instrumental, informational, and appraisal support. Expanding on this framework, Malecki and Demaray (2003) described TS as teachers’ demonstration of caring, understanding, and guidance, which facilitates students’ academic, social, and emotional development. To further conceptualize and assess TS, Doménech-Betoret et al. (2020) developed the Teacher Support Questionnaire based on The Educational Situation Quality Model (MOCSE, acronym in Spanish) framework (MOCSE-TSQ), which encompasses ten key dimensions, i.e., content comprehension support, which facilitates students’ understanding through clear explanations and multiple representations; teacher accessibility and closeness, which emphasizes emotional proximity and availability; autonomy support, which nurtures independent thinking and self-directed learning; peer support facilitation, which enhances both academic achievement and social skills development; interest enhancement, which focuses on triggering and maintaining situational interest; effort acknowledgment, which fosters growth mindset through specific praise; learning guidance, which provides structured scaffolding toward independence; self-competency support, which promotes mastery experiences and modeling; didactic resource provision, which ensures appropriate learning materials; and feedback provision, which addresses task, process, self-regulation, and self-level aspects of students’ learning.

Research has consistently shown that these dimensions of TS significantly predict students’ academic achievement, engagement, and long-term success in educational contexts (Allen et al., 2013; Clifton et al., 2004; Cornelius-White, 2007; Doménech-Betoret et al., 2020; Klem & Connell, 2004; Roorda et al., 2011; Wentzel et al., 2010; Gregory & Weinstein, 2004; DeGarmo & Martinez, 2006; Plunkett et al., 2008). Extending beyond general academic contexts, existing research shows that TS significantly influences musical performance achievement and skills through teacher-student interactions in shaping musical outcomes (Creech & Hallam, 2011; McPherson et al., 2006; Power & Powell, 2018).

Specifically, teachers play a critical role in helping students overcome learning barriers by providing essential resources and guidance while fostering a supportive, encouraging, and autonomy-promoting learning environment. This support enhances students’ sense of respect, engagement, and confidence, making them more willing to explore new techniques and performances (Bonneville-Roussy et al., 2020). However, direct evidence on the impact of teacher support (TS) on students’ level of piano skill (LPS), particularly among pre-service preschool teachers, remains limited. Therefore, this study proposes Hypothesis 1:

**H1.**  *Teacher support (TS) has a positive impact on the level of piano skill (LPS) of pre-service preschool teachers.*

### 1.2. The Mediating Role of Music Self-Efficacy

Self-efficacy refers to an individual’s confidence in their ability to accomplish a specific task, a confidence gradually formed through interactions between the individual and the social environment (Elliot & Dweck, 2005). Music self-efficacy (MSE) specifically denotes the positive beliefs musicians or learners hold regarding their capabilities in learning and performing music (Ritchie & Williamon, 2011). This belief not only influences an individual’s attitude and behavior when facing musical challenges but is also a crucial component of the music learning process (McCormick & McPherson, 2003). Students with strong MSE are more likely to set challenging goals, maintain focused practice routines, and actively seek constructive feedback to improve their performance skills (Miksza, 2015; Zelenak, 2024). This heightened sense of capability also contributes to reduced performance anxiety and increased willingness to participate in public performances, ultimately promoting more holistic musical development and sustained engagement in musical activities (McPherson & McCormick, 2006; Zelenak, 2024).

Research also indicates that learners’ MSE is significantly affected by external support, such as teacher feedback, instructional guidance, and supportive learning environments (Hendricks, 2014, 2016; Howe & Sloboda, 1991; Zelenak, 2015). Studies find that supportive teaching practices and positive instructor interactions significantly enhance learners’ confidence in their musical abilities (Federici & Skaalvik, 2014; Hendricks, 2016; Zelenak, 2015). For instance, Hendricks (2016) demonstrated that when music teachers construct supportive learning environments and offer constructive feedback, students develop stronger beliefs in their musical capabilities. These findings underscore the crucial role of teacher support in nurturing and sustaining students’ MSE.

The positive relationship between MSE and performance achievement has been well-documented in contemporary research. Evidence shows that students with higher levels of MSE achieve superior performance outcomes (McPherson et al., 2019; Zelenak, 2015, 2019). When students possess strong beliefs in their musical capabilities, they are more likely to engage in self-regulated practice and demonstrate resilience in their learning process (Varela et al., 2016; Miksza & Tan, 2015).

However, while the relationship between general TS and self-efficacy has been extensively studied, research specifically examining the mediating role between TS and LPS remains relatively limited. This gap is particularly notable for pre-service preschool teachers enrolled in teacher education programs, where research on this topic is scarce. Based on these findings, this study proposes Hypothesis 2:

**H2.**  *Music self-efficacy (MSE) mediates the relationship between teacher support (TS) and the level of piano skill (LPS) among pre-service preschool teachers.*

### 1.3. The Mediating Role of Learning Engagement

Learning engagement (LEG) refers to the time and energy learners invest in effective learning activities (Kuh, 2001). It encompasses learners’ willingness, participation, focus, and the resulting perceptions during the learning process (Pike et al., 2011). Specifically, it is reflected in learners’ sustained effort, determination, and perseverance when engaging in learning tasks. Extensive research has shown that TS positively influences LEG (Goodenow, 1993; Tas et al., 2019; Wentzel, 1997). For example, Goodenow (1993) suggested that when learners perceive high level of TS, they are more likely to work harder and persist in the face of difficulties. Wentzel (1997) found that TS promotes academic effort among students. Further research demonstrated that students’ perceptions of science TS had a positive influence on both their task value and academic self-concept in science (Tas et al., 2019). These findings underscore the significant impact of TS on students’ LEG, as evidenced through enhanced persistence, effort, and task value.

A substantial body of evidence has established that LEG plays a critical role in students’ academic success. The effort learners invest in is key to achieving high academic performance (Skinner et al., 1990), and LEG directly affects their achievements (Klem & Connell, 2004; De Villiers & Werner, 2016). In music education, research indicates that musical engagement significantly influences both esthetic experience and musical development and achievement (Wang & Huang, 2024). Therefore, understanding the factors that impact students’ LEG is essential for fostering successful learning outcomes and academic achievement.

Despite extensive research on the impact of TS on LEG, studies specifically focusing on how these factors influence LPS among pre-service preschool teachers remain limited. Therefore, this study proposes Hypothesis 3:

**H3.**  *Learning engagement (LEG) mediates the relationship between teacher support (TS) and the level of piano skill (LPS) among pre-service preschool teachers.*

### 1.4. The Chain Mediating Role of Music Self-Efficacy and Learning Engagement

The above studies indicate that MSE and LEG mediate the pathways through which TS affects LPS. Teachers are the most significant agents influencing learners’ motivation and behavior (Wentzel et al., 2016). TS can enhance learners’ self-efficacy, autonomy, and sense of belonging (Skinner et al., 2008).

**Figure 1:**
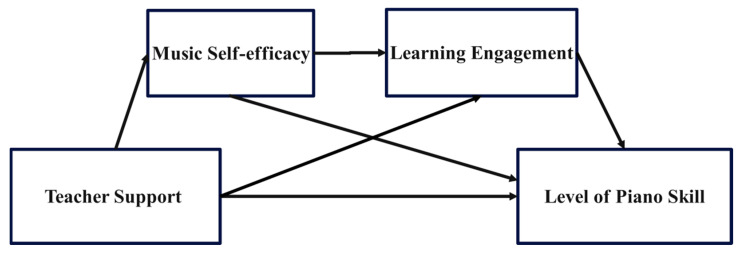
Hypothetical Model of the Chain Mediation in this Study.

Research has shown that low self-efficacy is associated with reduced interest and engagement in learning, whereas high self-efficacy fosters greater interest and a willingness to invest time and effort (Ardura & Galán, 2019). Learners with high self-efficacy are more likely to exhibit higher levels of engagement. This heightened self-efficacy encourages learners to set personal learning goals, approach academic challenges with a positive attitude, actively seek solutions, and maintain long-term commitment to their studies, thereby increasing their engagement (Chai et al., 2011; Cooper, 2014). The resulting increase in LEG ultimately enhances academic performance (Skinner et al., 1990). This sequential impact underscores the significance of both mediators in the chain-mediated relationship between TS and LPS. Based on the above analysis, this study proposes Hypothesis 4 (Figure 1).

**H4.**  *Music self-efficacy (MSE) and learning engagement (LEG) play a chain mediating role in the relationship between teacher support (TS) and the level of piano skill (LPS) of pre-service preschool teachers.*

## 2. Methods

### 2.1. Participants

The piano classes were conducted only for first, second, and third-year students, so fourth-year students were excluded from this study. A total of 444 questionnaires were distributed. After removing invalid responses based on predefined criteria, such as incomplete answers, identical responses to all questions, or patterns of answers, 430 valid questionnaires were retained. Questionnaires were distributed and collected immediately after completion in classroom settings, which achieved a 100% response rate. This approach ensured focused participation and minimized non-response bias. Of the participants, 44 were male (10.2%) and 386 were female (89.8%). Additionally, 195 participants identified as first-year students (45.3%), 138 as second-year students (32.1%), and 97 as third-year students (22.6%). Among them, 5 participants (1.2%) were 18 years old, 124 (28.8%) were 19, 148 (34.4%) were 20, 104 (24.2%) were 21, and 49 (11.4%) were 22. Given the educational system in China, most university students had completed preschool, primary, middle and high school education before entering universities. Typically, this education pathway includes three years preschool education, six years of primary education, three years of junior high school, and three years of senior high school.

The participants in this study were recruited through random cluster sampling of pre-service preschool teachers from the preschool teacher education program at a public normal university in Shanghai, China. The study utilized evaluation scores from curriculum-required public piano performances as the metric for the level of piano skill. These skill demonstrations, conducted within the piano courses of the preschool teacher education program. This study was reviewed and approved by the Ethics Committee of Shanghai Normal University (IRB#2024091). Participants were informed about the purpose of the questionnaire, confidentiality, and consent was obtained from all respondents.

### 2.2. Measures

#### 2.2.1. Teacher Support

Teacher support in the piano courses of the preschool teacher education program, as measured by the Teacher Support Questionnaire (MOCSE-TSQ). The Teacher Support Questionnaire (MOCSE-TSQ) was developed by Doménech-Betoret et al. (2020). The scale consists of 55 items corresponding to 10 dimensions: content comprehension support (7 items), teacher accessibility and closeness (8 items), autonomy support (7 items), peer support (5 items), awakening interest in the subject (5 items), acknowledging the students’ effort (5 items), guiding students in their learning (5 items), self-competency support (5 items), providing didactic resources (4 items), and teacher feedback (4 items). Respondents used a 6-point scale, ranging from 1 (totally disagree) to 6 (totally agree). Higher scores indicate higher levels of TS. The overall Cronbach’s alpha coefficient of Teacher Support Questionnaire (MOCSE-TSQ) in this study was 0.96, the coefficient of content comprehension support subscale was 0.80, the teacher accessibility and closeness subscale was 0.83, the autonomy support subscale was 0.65, the peer support subscale was 0.74, the awakening interest in the subject subscale was 0.74, the acknowledging the students’ effort subscale was 0.76, the guiding students in their learning subscale was 0.71, the self-competency support subscale was 0.72, the providing didactic resources subscale was 0.69, and the teacher feedback subscale was 0.65.

#### 2.2.2. Music Self-Efficacy

Music Self-efficacy in the piano courses of the preschool teacher education program, was assessed based on the Attitudes Toward Musical Activities and Performance, developed by Ritchie and Williamon (2011). Pre-service early childhood educators are required to demonstrate piano performance skills in kindergarten activities, using music to spark young learners’ curiosity and creativity. This process of piano playing requires performance skills and emotional expression that are comparable to those needed for public performances. We have selected this assessment tool (Ritchie & Williamon, 2011) to evaluate educators’ self-efficacy in the piano courses of the preschool teacher education program. The scale comprises 22 items corresponding to the two dimensions of self-efficacy for musical learning (11 items) and self-efficacy for musical performing (11 items). A 7-point scale was utilized, from 1 (much less ability) to 7 (excellent ability) with higher scores indicating greater levels of MSE. The overall Cronbach’s alpha coefficient of MSE was 0.92, the coefficients of self-efficacy for musical learning subscale was 0.85, and the self-efficacy for musical performing subscale was 0.86.

#### 2.2.3. Learning Engagement

Pre-service preschool teachers’ piano learning engagement in the piano courses of the preschool teacher education program, as measured by the Classroom Engagement Inventory in Music (CEI-M), developed by Joel E. Pagán (2018). The scale consists of 24 items corresponding to five dimensions: behavioral engagement-effortful class participation (5 items), behavioral engagement-compliance (3 items), affective engagement (5 items), cognitive engagement (5 items), and disengagement (6 items). A 5-point scale was utilized, from 1 (Never) to 5 (Each day of class). A higher score indicates a greater level of learner engagement in the music course. In this study, the Cronbach’s alpha coefficient of learning engagement was 0.92, the coefficients of behavioral engagement-effortful class participation subscale was 0.88, the behavioral engagement-compliance subscale was 0.85, the affective engagement subscale was 0.88, the cognitive engagement subscale was 0.91, the disengagement subscale was 0.60.

#### 2.2.4. Level of Piano Skill

In this study, the Level of Piano Skill (LPS) is evaluated by the examination-based assessment in the piano courses of the preschool teacher education program. This approach is grounded in the standardized evaluation framework adopted by teacher-training universities in Shanghai, where piano proficiency is systematically assessed through performance-based evaluations. As outlined in official guidelines (East China Normal University, 2024; Shanghai Normal University, 2024), these courses emphasize process-oriented assessment to holistically track skill. By adopting this localized model, our study integrates a competency-driven evaluation paradigm into the piano curriculum for pre-service preschool teachers. This method aligns with formative assessment principles, emphasizing continuous feedback and incremental skill refinement. It not only strengthens technical and pedagogical competencies but also bridges the gap between academic training and real-world classroom demands. The piano courses for first, second, and third-year students employ a standardized assessment criterion to maintain consistency and equity in the evaluation process. The assessment system includes three primary categories: technical skills, musical interpretation, and overall musical ability. Each category comprises distinct assessment criteria, amounting to ten standards, which together inform the final performance evaluation. All criteria are assessed using a 10-point scale, which allows for the classification of performance into three levels: low (scores of 1–3), medium (scores of 4–7), and high (scores of 8–10) for each criterion, as shown in Table 1.

The data reveal that within each category, i.e., technical skills, musical interpretation, and comprehensive musical ability, students’ strengths and weaknesses are distinctly identifiable (Refer to the columns on the right in Table 2, which represent the sums of the category types per student). This Table 2 clearly illustrates students’ piano performance scores, reflecting their overall piano proficiency. By visualizing multidimensional proficiency data, Table 2 not only validates the utility of our proposed evaluation framework but also establishes a foundation for subsequent empirical analysis of teacher support strategies. Specifically, it sets the stage for exploring how educators might perceive these insights. Through the analysis of detailed scores across various criteria, educators can identify specific areas for student improvement.

### 2.3. Research Procedures and Data Processing

The questionnaire was distributed anonymously to the classes, following a uniform protocol. Each questionnaire contained a unique tracking number that matched pre-assigned student identifiers, enabling confidential data linkage while preserving anonymity. Participants completed the questionnaires in approximately 20 min, with results gathered on-site. The data derived from these questionnaires were collated and analyzed using SPSS 26.0. The mediating effects of MSE and LEG on the relationship between TS and LPS were examined using model 6 of the PROCESS plug-in for the SPSS macro program developed by Hayes (Preacher & Hayes, 2004).

### 2.4. Statistical Analysis

The study utilized Harman’s one-factor test, Pearson’s correlations, t-tests, and mediation models to analyze the data. All data collection and analysis were executed using SPSS 26.0 software. To assess potential common method bias, Harman’s one-factor test was applied. Descriptive statistics for key variables—teacher support, music self-efficacy, learning engagement, and level of piano skill—were presented through means and standard deviations. The relationships among these variables were examined using Pearson’s product–moment correlation coefficient. Independent samples t-tests were conducted to explore gender differences, while a one-way ANOVA was used to evaluate variations in different variables across various grade levels. Furthermore, a bootstrapping procedure with 5000 resamples was employed to estimate the 95% confidence interval for the mediating effect within the model.

## 3. Results

### 3.1. Common Method Bias Test

This study employed Harman’s one-factor test, indicating the presence of 19 components with eigenvalues greater than 1. The first factor accounted for 20.52% of the variability, which is below the critical threshold of 40% (Hair et al., 1998). The findings indicate that common technique variance was not a significant concern in this research.

### 3.2. Descriptive Statistics and Correlation Analysis for Each Variable

This study utilized Pearson correlation analysis to examine the relationships between variables, identifying significant correlations. The results showed that TS was significantly and positively correlated with MSE (r = 0.22, *p* < 0.01), LEG (r = 0.17, *p* < 0.01), LPS (r = 0.19, *p* < 0.01). Additionally, MSE showed a significant positive correlation with LEG (r = 0.20, *p* < 0.01), LPS (r = 0.21, *p* < 0.01). Furthermore, LEG demonstrated a significant positive correlation with LPS (r = 0.27, *p* < 0.01). Moreover, a significant positive correlation was observed between gender and LPS (r = 0.25, *p* < 0.01). Detailed results are presented in Table 3.

Furthermore, results of independent samples t-tests (Table 4) indicated that the LPS among female students (M = 84.15) are significantly higher than those male students (M = 79.84), *p* < 0.001. A one-way ANOVA revealed significant differences in MSE and LEG across different grade levels, *p* < 0.01, as shown in Table 5.

### 3.3. Mediator Model

The study initially assessed the importance of the regression coefficients for three pathways in sequence, i.e., teacher support → music self-efficacy → learning engagement → level of piano skill, music self-efficacy → level of piano skill, teacher support → learning engagement and teacher support → level of piano skill. Then, the study analyzed the chain mediation effect of these variables using the PROCESS macro program in SPSS. In this analysis, TS was considered the independent variable, MSE and LEG were treated as mediator variables, and LPS was the dependent variable.

The results of the regression analyses (Table 6) revealed that TS positively predicted MSE (β = 0.22, *p* < 0.001). MSE had a significant positive predictive impact on LEG (β = 0.17, *p* < 0.001), which was also a positive predictor of LPS (β = 0.22, *p* < 0.001). Furthermore, MSE was a significant positive predictor of LPS (β = 0.14, *p* < 0.01). TS positively predicted LEG (β = 0.13, *p* < 0.01). TS was found to be a significant positive predictor of LPS (β = 0.12, *p* < 0.05), supporting Hypothesis 1.

**Figure 2:**
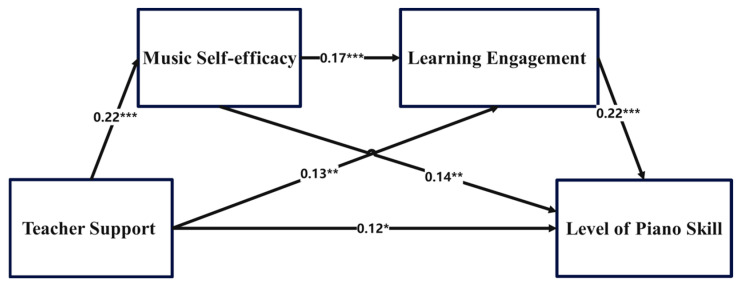
Mediation Model Between Teacher Support and Level of Piano Skill. Note. * *p* < 0.05; ** *p* < 0.01; *** *p* < 0.001.

Following Hayes’s recommendation, we implemented the bootstrap procedure by performing the mediation analysis on 5000 randomly generated resamples of the data and then computing the 95% confidence interval for the mediating effect from the distribution of the resulting estimates, as this confidence interval that excludes zero signifies the conclusion that the indirect effect is positive (Hayes, 2017). The results revealed that the mediating role of MSE and LEG was significant in TS and LPS. As shown in Table 7 and Figure 2, the size of this total indirect effect was 0.47, 95% CI = [0.2333, 0.7320], and the ratio of the mediating effect to the total effect (1.33) was 35.34%. Specifically, the value of the indirect effect of a single mediated path for MSE was 0.21 (95% CI = [0.0404, 0.4167], ratio to total effect = 15.79%), thus confirming Hypothesis 2. The value of the indirect effect of a single mediated path for LEG was 0.20 (95% CI = [0.0534, 0.3812], ratio to total effect = 15.04%), validating Hypothesis 3. The value of the indirect effect of a chained mediated path for MSE and LEG was 0.06 (95% CI = [0.0220, 0.1063], ratio to total effect = 4.51%), validating Hypothesis 4. The 95% confidence intervals for the indirect effects of all three paths do not contain zero; therefore, the mediating effect of all three paths is significant.

## 4. Discussion, Implications and Limitations

To explore the underlying mechanisms through which TS influences LPS in pre-service preschool teachers within preschool teacher education programs, our study developed a chain mediation model to examine the sequential mediating effects of MSE and LEG. Our findings indicate that TS is positively associated with MSE, which, in turn, positively influences LEG. The relationship between TS and LPS in pre-service preschool teachers is mediated through both MSE and LEG. Additionally, our results demonstrate that TS predicts LPS via the sequential mediation of MSE and LEG. This study not only enhances the understanding of how TS impacts the LPS of pre-service preschool teachers but also provides deeper insights into the complex relationships between social support, individual psychological traits, and musical skill outcomes. Burak (2019) proposed that the perception of pre-service preschool teachers regarding the sufficiency and quality of their education is a predictive factor for their low sense of music self-efficacy. Burak pointed out that playing an instrument alone should not be regarded as the sole factor in explaining the relationship between music knowledge, skills and self-efficacy; the aspects of communication, interaction and relationships in music education should also be explored. Although there is relatively little research on the relationship between music skills and social support for pre-service preschool teachers, Orejudo et al. (2021) taking students of different stages of music learning as the research subjects, concluded that social support has a significant relationship with music self-efficacy, it was found that social support has a significant positive correlation with music self-efficacy.

Building on this perspective, teacher support—as a critical form of social support—constitutes a direct and proximal learning support system that profoundly shapes students’ educational experiences and outcomes. This study posits teacher support as the independent variable stems from its role as the most direct and actionable form of social support within learning environments. The significance of teacher support as an independent variable is further underscored by its immediate impact on learners within educational settings. Unlike other forms of social support that might be more indirect or distal (such as peer support or family support), teacher support is uniquely positioned to provide timely and relevant assistance that aligns closely with curriculum objectives and individual student needs. By acting as a bridge between students and the resources they need, teachers play an indispensable role in facilitating learning and fostering positive educational outcomes. Given its immediacy in instructional settings, teacher support encompasses not only emotional encouragement but also scaffolding in skill development and access to resources, which may directly reinforce learners’ self-efficacy beliefs. This study not only enhances the understanding of how teacher support impacts the level of piano skill of pre-service preschool teachers but also provides deeper insights into the complex relationships between social support, individual psychological traits, and musical skill outcomes. These findings offer valuable guidance for developing targeted interventions to improve the piano skills of pre-service preschool teachers.

### 4.1. Discussion

#### 4.1.1. Teacher Support Significantly Predicts Level of Piano Skill

Consistent with previous research, this study found that TS significantly and positively predicts LPS, which suggests that TS can enhance students’ sense of security and confidence, motivating them to dedicate more time to musical training, thus improving performance (Wentzel et al., 2010). TS is a critical factor in the learning progress of music students (Moore et al., 2003) and is strongly associated with musical performance achievement (Nogaj & Ossowski, 2015). Pre-service preschool teachers are typically adults and often beginners in piano (Iwaguchi, 2012). Some researchers have noted that, compared to children, adult piano learners are more prone to frustration, as their cognitive and motor skills may not be as well coordinated (Wristen, 2006).

Therefore, external intervention by teachers becomes particularly important in the process of learning piano skills. The professional qualities of teachers are essential factors in promoting learner’s learning progress (Upitis et al., 2017). Self-determination theory suggests that learners’ performance improves when they receive support in competence, relatedness, and autonomy from teachers (Filippello et al., 2020; Oriol-Granado et al., 2017; Zhao & Qin, 2021). Specifically, encouragement and understanding boost learners’ confidence, reduce anxiety, and help direct their focus toward skill development.

#### 4.1.2. Mediating Role of Music Self-Efficacy

In social cognitive theory, the environment, cognition, and behavior are reciprocal influences (Bandura, 1986). Based on this framework, previous research has shown that when learners perceive TS, their self-efficacy is enhanced (Kim et al., 2018). Further studies have highlighted the close relationships between TS, MSE, and musical skill development in music education (Orejudo et al., 2021; Zelenak, 2020; Zarza et al., 2020). This mediating effect supports previous findings that TS enhances self-efficacy, which, in turn, promotes learning outcomes (Ryan & Deci, 2000; Zarza et al., 2020).

The present study extends these findings by demonstrating that MSE significantly mediates the relationship between TS and LPS among pre-service preschool teachers. This mediating effect aligns with prior research indicating that TS strengthens self-efficacy, which in turn fosters improved learning outcomes (Ryan & Deci, 2000; Zarza et al., 2020). The consistency of this relationship across diverse demographic groups and musical instruments suggests that self-efficacy’s impact on musical achievement is both stable and generalizable. For instance, McCormick and McPherson (2003) examined 332 students aged 9 to 18 learning to play either piano, string, brass, or woodwind instruments and found that self-efficacy was the best predictor of actual performance outcomes. Furthermore, McPherson and McCormick (2006) conducted a comprehensive study involving 686 participants—446 females (65%) and 240 males (35%)—who participated in piano performance exams administered by the Australian Music Examinations Board (AMEB). This research concluded that self-efficacy significantly influences music achievement, thereby reinforcing its critical role across different demographics and musical contexts. These studies, among others, address the relationship between self-efficacy and music achievement across various ages and types of instruments, all demonstrating that self-efficacy is a significant predictor of music achievement. Additional support for this finding comes from other research, such as Ritchie and Williamon (2011) and Zelenak (2019), which further corroborates the impact of self-efficacy on music achievement. Learners with strong self-efficacy exhibit greater engagement in their studies and maintain confidence in their ability to reach their goals, leading to enhanced educational outcomes (Ryan & Deci, 2000). In the context of music education, the quality and perception of teacher instruction emerge as crucial factors in developing pre-service preschool teachers’ MSE (Burak, 2019), which serves as a reliable predictor of musical performance outcomes (Zarza et al., 2020).

In the learning process, the support provided by teachers can enhance learners’ MSE. Music instructors can enhance students’ musical outcomes by promoting MSE (Zelenak, 2020). Pre-service preschool teachers may form negative beliefs regarding their MSE influenced by a range of internal and external factors (Burak, 2019). Pre-service preschool teachers encounter notable challenges in music education. Insufficient foundational music knowledge is prevalent.

#### 4.1.3. Mediating Role of Learning Engagement

According to Connell’s self-system theory, LEG is a malleable process influenced by both environmental and individual factors. Among these, learners’ perception of TS is a key variable affecting their LEG (Connell & Wellborn, 1991). Wentzel’s (1997) research indicates that TS significantly promotes learner engagement. When learners perceive TS, they exhibit greater effort and stronger perseverance when facing challenging learning tasks (Clifton et al., 2004; Zarza et al., 2020). Enhanced LEG serves as an effective conduit for influencing individual learning outcomes and personal growth (Connell & Wellborn, 1991). This study delves into the mediating role of LEG within the relationship between TS and improvements in piano skills. The findings reveal a robust positive correlation between learners’ perceptions of TS and their level of engagement (Brewster & Bowen, 2004; Garcia-Reid et al., 2005, 2015; Perry et al., 2010; Wentzel, 1998; Woolley & Bowen, 2007; Zarza et al., 2020) indicating that the more support teachers provide, the higher the level of engagement among learners.

This finding not only validates previous research conclusions (Descals-Tomás et al., 2021) but also elucidates the specific mechanism through which TS enhances LEG, thereby fostering improvements in piano skills. TS not only directly motivates learners but also indirectly promotes skill development by increasing engagement levels, establishing a positive feedback loop that drives continuous progress. The study highlights multiple challenges pre-service preschool teachers encounter in piano learning. From an external perspective, current preschool teacher education programs often fail to adequately prioritize music education, particularly in the teaching of instruments like the piano (Russell-Bowie, 2010). This lack of emphasis limits opportunities for acquiring necessary musical knowledge and skills and can indirectly diminish interest and confidence. Internally, adult learners like pre-service teachers face unique challenges due to their physiological and psychological characteristics. Adults generally learn at a slower pace, especially with skills requiring extensive practice, such as playing the piano. Moreover, limited time and resources allocated to music courses in teacher education programs make it difficult for learners to achieve significant progress solely through minimal in-class practice. Therefore, TS plays a pivotal role in this context.

#### 4.1.4. Chain Mediation of Music Self-Efficacy and Learning Engagement

Although previous research has indicated that social support influences the MSE and musical skills of music learners (Upitis et al., 2017), this study further reveals the chain mediating role of MSE and LEG in the relationship between TS and LPS. The decisions made by music learners regarding their learning and performance are shaped by personal experiences, the surrounding environment, and intrinsic beliefs (Ritchie & Williamon, 2011). For pre-service preschool teachers, who are typically piano beginners, TS thus enhances both MSE and LEG, effectively promoting piano skill development. These results align with prior research suggesting that meeting learners’ basic psychological needs results in more positive attitudes, courage to tackle difficulties, and high enthusiasm for learning (Kimbark et al., 2016). Conversely, if learners do not gain intrinsic confidence and satisfaction during skill acquisition, their enthusiasm may wane, leading to stagnation or decline in skill levels. Therefore, good MSE and LEG are crucial for the impact of TS on piano skills. In training pre-service preschool teachers, cultivating these aspects is key to enhancing piano proficiency.

This study clarifies how TS affects the piano skills of pre-service preschool teachers, highlighting the contributions of external and internal factors. It provides insights for addressing musical skill issues in teacher education programs, validating relationships between TS, MSE, LEG, and LPS. The mediating effect of MSE and LEG in the impact of TS on LPS reveals the intrinsic connection between psychological motivation and behavioral reinforcement. Based on the “support–feedback–reflection” cyclical framework, teachers can implement effective intervention through a three-stage progressive strategy: in the support stage, provide personalized guidance and progressive task design to establish initial confidence of ‘I can do it’; in the feedback stage, use intelligent systems to capture real-time performance during playing, and combine teacher-student interaction to identify key nodes for skill improvement; and in the reflection stage, guide learners to transform technical training into educational practice ability through teaching scenario simulation and peer evaluation. This model enables piano courses to break away from mere skill training and form a closed loop of ‘psychological empowerment—skill refinement—educational transfer’, helping prospective teachers simultaneously enhance their professional qualities and teaching competence. This study confirms the correlation among TS, pre-service preschool teachers’ MSE, LEG, and LPS within preschool teacher education programs’ piano courses. It further confirms the mediating role of MSE and LEG in the impact of TS on the LPS. These findings provide empirical evidence for the curriculum design and teaching improvements in piano courses for pre-service preschool teachers.

Furthermore, this study revealed that female students demonstrated significantly higher piano skill proficiency scores compared to their male counterparts. This disparity may be attributed to two interrelated factors. First, the cohort exhibited a pronounced gender imbalance, with male trainees constituting a minority within the predominantly female students. In such environments, male participants often feel inhibited when performing piano skills in front of predominantly female peers, exacerbated by the absence of same-gender peers for observational learning and peer modeling.

Additionally, third-year students displayed notably lower scores in both music self-efficacy and learning engagement. This decline likely coincides with their transition into mandatory educational internships in Shanghai and heightened prioritization of theoretical coursework in early childhood education to prepare for professional roles. These dual academic and practical commitments may inadvertently reduce opportunities for sustained musical practice and psychological investment in piano skill refinement during their final training phase.

### 4.2. Implications

Research has found a positive effect of teacher support on students’ piano skill learning. Therefore, competence support, which includes systematic training and professional guidance, assists in overcoming technical challenges and improving performance. Autonomy support fosters independent exploration, thereby enhancing intrinsic motivation and nurturing creativity. Effective TS in competence, relatedness, and autonomy not only increases learners’ motivation and confidence but also leads to significant improvements in their piano skills.

The study advocates the enhancement of Teacher Support for students in preschool teacher education programs. Specifically, curriculum and instructions should cultivate autonomous learning, emphasize music education’s significance, and foster confidence in musical competencies. Teachers can alleviate learning impediments and mitigate stress through personalized guidance, regular feedback, and support (Perkins et al., 2017). Furthermore, establishing a positive and collaborative learning environment promotes students’ MSE through mutual learning and experience sharing. Teachers should prioritize both professional knowledge dissemination and the emotional development of trainees, thereby igniting their enthusiasm and confidence in music instruction.

The research also indicates that teacher support can take various forms, including providing rich learning materials, creating an inclusive and encouraging environment, and offering timely and effective feedback and guidance. For practical courses like piano instruction, frequent teacher-student interactions are essential. These interactions help resolve technical issues, stimulate creativity, and enhance enthusiasm and motivation for music learning.

The research results indicate that music self-efficacy and learning engagement as mediating variables reveal the internal mechanism by which teacher support affects piano skills. The “support–feedback–reflection” cycle framework provides a structured teaching method; in the initial support stage, teachers establish students’ foundational confidence by designing progressive learning tasks and providing personalized guidance. The subsequent feedback process requires the integration of technical evaluation tools and instant interaction to accurately identify key nodes in skill development. The final reflection stage promotes students to transform music skills into educational and teaching abilities through situational simulations and practical exercises. This teaching model breaks the limitations of traditional piano teaching and constructs a complete development path from “psychological supports” to “skill improvement” and then to “educational application”. Through this integration method, piano teaching is no longer just a simple skill training, but has become an important way to cultivate the core professional qualities of pre-service early childhood education teachers, enabling them to more effectively use music to promote the comprehensive development of young children in future educational practices.

### 4.3. Limitations

Despite the valuable insights gained, certain limitations exist. First, due to cross-sectional research design, the study may not reveal true causal relationships. Future research should employ longitudinal cross-lagged studies or experimental designs to validate the causal relationship between teachers’ teaching practice and learning achievement. Second, the quantitative analysis in this study was based on student feedback only, which may not have provided a sufficiently detailed and deeper understanding of how students learn piano playing skills. Third, the 101-item survey design, while comprehensive, may have inadvertently contributed to respondent fatigue due to its length and the relatively low Cronbach’s alpha values for certain subscales may reflect challenges in cross-cultural adaptation of the translated questionnaire. While rigorous translation and validation protocols were followed, linguistic nuances between the original English instrument and its Chinese adaptation could have introduced ambiguities that affected participants’ consistent interpretation of specific items, thus leading to reduced consistency. However, during the research, the translated Chinese version of the questionnaire was carefully reviewed by the researchers, who confirmed that the items were interpretable to participants. This limitation underscores if this instrument is reused, further consideration of its cultural adaptability will be necessary. The revision acknowledges the measurement limitation while contextualizing it within the broader methodological challenges of cross-cultural instrumentation. Fourth, regarding the selection of the MSE scale, this study adopts the instrument developed by Ritchie and Williamon (2011), originally designed for conservatory and collegiate music students. However, given that the piano performance requirements in early childhood educator training programs—specifically demonstrating technical proficiency and emotional expressiveness during kindergarten activities to stimulate children’s creative engagement—demand competencies comparable to those expected in public performances, this adaptation was considered methodologically appropriate. Fifth, we also recognize the possibility that there may be more robust assessment frameworks for measuring piano-learning self-efficacy among pre-service early childhood educators within specialized teacher preparation programs. Therefore, mixed-methods research is necessary to further validate the relationships among the variables. Lastly, in addition to teaching practice, other external environmental factors such as social support, school support, and family support are also critical and may significantly influence the piano performance achievements of pre-service preschool teachers. For example, exploring how societal views on piano teaching in early childhood education impact the learning behaviors of pre-service preschool teachers in educational programs could provide valuable insights.

## 5. Conclusions

Overall, this study provides valuable insights that inform piano instruction for pre-service preschool teachers by insights that may inform piano instruction for pre-service preschool teachers by uncovering the mechanism through which teacher support positively predicts piano ability levels via the chain-mediated effects of musical self-efficacy and learning engagement among pre-service preschool educators. In response to the call for more research on the skill sets of early childhood education teachers (Burak, 2019), this study establishes a new “support–feedback–reflection” pathway, leveraging teacher support and its chain-mediated effects on musical self-efficacy and learning engagement. This pathway serves as a reference point for enhancing educational practices from psychological and behavioral perspectives, particularly focusing on teacher support (Doménech-Betoret et al., 2020). For pre-service preschool teachers who are beginners in piano, this approach can significantly enhance their learning experiences within teacher education programs. In preschool teacher education programs, piano teachers could potentially enhance students’ learning experiences by focusing on building piano confidence and skills, while considering approaches that foster relatedness, offer clear guidance, encourage autonomous learning, and incorporate individualized instruction where feasible. Future research may explore which teaching factors may have positive effects in specific educational contexts.

## Figures and Tables

**Table 1:** Evaluation Rubrics of Students’ Level of Piano Skill.

Criterion	Description	Category
1	Skill in performing accurately and with clarity.	Technical Skills
2	Skill in performing a range of techniques with control and fluency.	Technical Skills
3	Skill in expressive communication through articulation and phrasing.	Musical Interpretation
4	Skill in performing with musicality through creativity and individuality.	Musical Interpretation
5	Skill in maintaining consistent and accurate rhythmic patterns throughout the performance.	Musical Interpretation
6	Skill in producing a range of dynamic contrasts to enhance the musical expression.	Musical Interpretation
7	Skill in producing a range of expressive tonal qualities appropriate to the musical context.	Comprehensive Musical Ability
8	Skill in controlling and maintaining an appropriate tempo throughout the performance.	Comprehensive Musical Ability
9	Skill in performing scales, arpeggios, and chords smoothly and accurately.	Technical Skills
10	Skill in presenting a musical program within appropriate performance conventions, demonstrating an informed interpretation of a range of styles and the ability to deliver overall performance that encompasses comprehensive musical skills.	Comprehensive Musical Ability

**Table 2:** Performance Scores of Individual Student Samples.

Samples	1	2	3	4	5	6	7	8	9	10	Grades (SUM)
	Technical Skills	Technical Skills	Musical Interpretation	Musical Interpretation	Musical Interpretation	Musical Interpretation	Comprehensive Musical Ability	Comprehensive Musical Ability	Technical Skills	Comprehensive Musical Ability	
student	6	7	7	8	8	6	7	8	6	8	71
student	9	9	8	9	8	9	9	9	7	8	85
student	9	8	8	8	9	7	9	8	9	9	84
student	7	6	9	9	8	9	9	8	9	9	83

**Table 3:** Results of Descriptive Analysis and Inter-correlations Among Variables.

	1	2	3	4	5	6
1. Gender	1.00					
2. Grade	0.00	1.00				
3. Teacher support	0.03	0.00	1.00			
4. Music self-efficacy	−0.02	−0.08	0.22 **	1.00		
5. Learning engagement	0.08	−0.15 **	0.17 **	0.20 **	1.00	
6. Level of piano skill	0.25 **	0.02	0.19 **	0.21 **	0.27 **	1.00
M	1.90	1.77	5.32	4.44	3.99	83.70
SD	0.30	0.79	0.75	1.17	0.63	5.28

Note. ** *p* < 0.01.

**Table 4:** Mean, Standard Deviation and Result of T-tests in Variables by Gender.

Variables	Male (N = 44)	Female (N = 386)	t
LPS	79.84 ± 6.01	84.15 ± 5.02	−4.57 ***
TS	5.25 ± 0.69	5.32 ± 0.75	−0.62
MSE	4.50 ± 1.27	4.43 ± 1.16	−0.38
LEG	3.84 ± 0.75	4.01 ± 0.62	−1.64

Note. *** *p* < 0.001. LPS = level of piano skill; TS = teacher support; MSE = music self-efficacy; LEG = learning engagement.

**Table 5:** Results of ANOVA Tests for Grade-Level Differences Across Variable.

Grade	LPS		TS		MSE		LEG	
	M	SD	M	SD	M	SD	M	SD
First-year students	83.46	5.33	5.29	0.74	4.43	1.21	4.06	0.63
Second-year students	84.14	5.29	5.38	0.73	4.69	1.27	4.02	0.61
Third-year students	83.58	5.21	5.27	0.78	4.09	0.84	3.80	0.64
F	0.70		0.87		7.58 **		5.57 **	
LSD					3 < 1, 2		3 < 2, 1	

Note. ** *p* < 0.01. LPS = level of piano skill; TS = teacher support; MSE = music self-efficacy; LEG = learning engagement.

**Table 6:** Regression Results of Variables in the Models.

Regression Equation		Overall Fitting Index			Significance of Regression Coefficients	
Outcome Variables	Predictive Variables	R	R^2^	F	β	t
MSE	TS	0.22	0.05	21.19	0.22	4.60 ***
LEG	TS	0.24	0.06	12.80	0.13	2.66 **
	MSE				0.17	3.62 ***
LPS	TS	0.33	0.11	17.78	0.12	2.57 *
	MSE				0.14	2.93 **
	LEG				0.22	4.69 ***

Note. *** *p* < 0.001; ** *p* < 0.01; * *p* < 0.05. LPS = level of piano skill; TS = teacher support; MSE = music self-efficacy; LEG = learning engagement.

**Table 7:** Standardized Total, Direct and Indirect Effects, and 95% Confidence Intervals.

Pathway	Estimate	SE	Ratio	95% Confidence Interval	
				Lower	Upper
TS-MSE-LPS	0.21	0.10	15.79%	0.04	0.42
TS-LEG-LPS	0.20	0.08	15.04%	0.05	0.38
TS-MSE-LEG-LPS	0.06	0.02	4.51%	0.02	0.11
Total indirect effect	0.47	0.13	35.34%	0.23	0.73
Direct effect	0.86	0.33	64.66%	0.01	0.20
Total effect	1.33	0.34	100.00%	0.00	0.67

Note. LPS = level of piano skill; TS = teacher support; MSE = music self-efficacy; LEG = learning engagement.

## Data Availability

The data presented in this study is available on request from the corresponding author. Data are not publicly available due to privacy or ethical restrictions.
